# Personalizing Evaluation and Care in Alzheimer's Disease: Tackling the Challenges of clinical presentation

**DOI:** 10.1002/alz70860_103694

**Published:** 2025-12-23

**Authors:** Thomas TANNOU, Sonia BÉLANGER, Paolo Vitali, Felix Pageau, Ziad Nasreddine, Marie Christine Le Bourdais, Guy Lacombe

**Affiliations:** ^1^ CRIUGM, CIUSS Centre‐Sud de l'Ile de Montréal, Montreal, QC, Canada; ^2^ Ministre des Ainés ‐ Ministère de la Santé et des Services sociaux, Quebec, QC, Canada; ^3^ The McGill University Research Centre for Studies in Aging, Montreal, QC, Canada; ^4^ Université Laval, Québec, QC, Canada; ^5^ MoCA Clinic and Institute, Greenfield Park, QC, Canada; ^6^ Alzheimer Society of Montreal, Montreal, QC, Canada; ^7^ CIUSSS de l'Estrie‐CHUS, Sherbrooke, QC, Canada

## Abstract

How can individuals adhere to an intervention plan or access health and social care services if they are unaware of their need for assistance? Anosognosia, defined as the lack of awareness of one's pathological condition, affects 20–40% of people living with major neurocognitive disorders (MNCD), such as Alzheimer's disease, even in its early stages. Rooted in memory impairments and prefrontal brain region dysfunction, anosognosia often causes individuals to overestimate their abilities, identify with a prior state of health, and oppose proposed interventions. This opposition undermines care plans, exhausts caregivers, and frequently results in crises such as unsafe behaviors, avoidable hospitalization, or institutionalization.

Multidimensional assessments play a critical role in addressing this complexity. Through neuropsychological testing, caregiver‐reported measures, neuroimaging (MRI, PET), and biomarkers, it's possible to identify key deficits that influence opposition to care and limit the effectiveness of standard and adapted interventions. Importantly, our studies demonstrated that anosognosia leads to impaired risk awareness and decision‐making, rooted in prefrontal and limbic brain dysfunction. For this reason, Alzheimer's disease should be seen more as a “adaptability disease” than a “memory disease”. These findings underline the urgent need for standardized evaluation tools and practices that are sensitive to these specific deficits.

Our research can support innovative care pathways that prioritize adaptability by analyzing the discourse of individuals with major neurocognitive disorders (MNCD). This analysis reveals patterns of overestimation of functional abilities, which lead to increased safety risks and care refusal. Our work underscores the importance of co‐designed care strategies that leverage technology—such as sensor‐based monitoring systems and AI tools—to dynamically address risk management. Additionally, insights into how intersectoral collaboration, guided by frameworks such as Quebec's Alzheimer Plan and policy, can bridge gaps between clinical research and real‐world care implementation will be shared.

Ultimately, offering actionable recommendations can enhance the assessment and care of individuals with Alzheimer's disease, while emphasizing the integration of cutting‐edge research into evolving, person‐centered practices.

